# Nationwide prevalence of human papillomavirus infection and viral genotype distribution in 37 cities in China

**DOI:** 10.1186/s12879-015-0998-5

**Published:** 2015-07-04

**Authors:** Rong Wang, Xiao-lei Guo, G. Bea. A. Wisman, Ed Schuuring, Wen-feng Wang, Zheng-yu Zeng, Hong Zhu, Shang-wei Wu

**Affiliations:** Division of Clinical Microbiology, School of Laboratory Medicine, Tianjin Medical University, Tianjin, China; Department of Microbiology, Kingmed Diagnostics, Guangzhou, China; Department of Gynecologic Oncology, University of Groningen, University Medical Center Groningen, Groningen, The Netherlands; Department of Pathology, University of Groningen, University Medical Center Groningen, Groningen, The Netherlands; Department of Epidemiology & Biostatistics, Tianjin Medical University, Tianjin, China

**Keywords:** Human papillomavirus, hrHPV prevalence, HPV genotyping, Cervical cancer, China

## Abstract

**Background:**

Type-specific high-risk HPV (hrHPV) infection is related to cervical carcinogenesis. The prevalence of hrHPV infection varies geographically, which might reflect the epidemiological characteristics of cervical cancer among different populations. To establish a foundation for HPV-based screening and vaccination programs in China, we investigated the most recent HPV prevalence and genotypic distributions in different female age groups and geographical regions in China.

**Methods:**

In 2012, a total of 120,772 liquid-based cytological samples from women enrolled for population- or employee-based cervical screening in 37 Chinese cities were obtained by the Laboratory of Molecular Infectious Diseases of Guangzhou KingMed. A total of 111,131 samples were tested by Hybrid Capture II and the other 9,641 were genotyped using the Tellgenplex™ HPV DNA Assay.

**Results:**

The total positive rate for hrHPV was 21.07 %, which ranged from 18.42 % (Nanchang) to 31.94 % (Haikou) and varied by region. The regions of Nanchang, Changsha, Hangzhou, Chengdu, Fuzhou, Guangdong, and Guiyang could be considered the low prevalence regions. Age-specific prevalence showed a “two-peak” pattern, with the youngest age group (15–19 years) presenting the highest hrHPV infection rate (30.55 %), followed by a second peak for the 50–60-year-old group. Overall, the most prevalent genotypes were HPV16 (4.82 %) and HPV52 (4.52 %), followed by HPV58 (2.74 %). Two genotypes HPV6 (4.01 %) and HPV11 (2.29 %) were predominant in the low-risk HPV (lrHPV) type, while the mixed genotypes HPV16 + 52 and HPV52 + 58 were most common in women with multiple infections.

**Conclusions:**

This study shows that HPV infection in China has increased to the level of an “HPV-heavy-burden” zone in certain regions, with prevalence varying significantly among different ages and regions. Data from this study represent the most current survey of the nationwide prevalence of HPV infection in China, and can serve as valuable reference to guide nationwide cervical cancer screening and HPV vaccination programs.

**Electronic supplementary material:**

The online version of this article (doi:10.1186/s12879-015-0998-5) contains supplementary material, which is available to authorized users.

## Background

HPV infection can cause a variety of genital diseases, and type-specific persistent infection of high-risk HPV (hrHPV) cases is strongly associated with cervical carcinogenesis [1]. Cervical cancer is the third most common type of cancer in women worldwide [2]. Effective implementation of cervical screening programs in developed countries has resulted in a steady reduction in the incidence of cervical cancer [3]. However, in China, the most populous country, cervical cancer remains the second leading cause of cancer deaths among 15- to 44-year-old females [4]. It is estimated that 75,434 women are diagnosed with cervical cancer annually (11.3/100,000) and 33,914 (45.0 %) of those women die because of cervical cancer [5, 6].

To date, more than 200 HPV genotypes have been identified, and ~40 HPV genotypes have been detected in the female genital tract. HPV16 and HPV18 are well known as oncogenic genotypes; additionally, HPV31, HPV33, HPV35, HPV39, HPV45, HPV51, HPV52, HPV56, HPV58, HPV59, HPV68, HPV69, and HPV 82 are also closely associated with cervical cancer. Therefore, all of these genotypes are classified as “high-risk” HPV. Meanwhile, “low-risk” genotypes, including HPV6, HPV11, HPV42, HPV43, and HPV44 are the causative agents for benign or low-grade changes in cervical cells, such as genital warts [7, 8].

The current hrHPV detection is supposed to serve as an additional approach for the early diagnosis of cervical cancer, and was implemented to complement less sensitive and non-objective cytology-based methods [3]. The high negative predictive value of hrHPV testing is applicable for the indication of a low-risk population, in which the cervical cancer screening interval can be safely extended [3]. Based on the results of clinical trials, several European countries will implement hrHPV testing as the primary screening modality [9]. In addition to HPV screening, HPV vaccination has been shown to be an effective strategy against HPV infection and has been recently implemented in most western countries. Although Cervarix (HPV16/18) and Gardasil (HPV6/11/16/18) protect against infection by HPV16 and HPV18, these vaccines provide no effect on some of the hrHPV types found in at least 25 % of cervical cancers [10]. Furthermore, the role of non-vaccine HPV types in the development of lesions remains unknown, and it remains possible that non-vaccine HPV types could replace these vaccine types as the causative agents for cervical precancerous lesions and cancer in vaccinated cohorts without sufficiently broad cross-protection [10].

The prevalence of HPV infection and type-specific distribution vary greatly both between nations [11], and between different regions within countries [12]. Additionally, several other risk factors can influence the prevalence of HPV, including genetic variation, sexual behavior (age at first sexual intercourse and individuals with multiple sex partners), biological predisposition of the immature cervix, and immunodeficiency [13]. Hence, surveillance of the general population is needed to assess the clinical benefits of screening and vaccination strategies.

Obtaining large-scale information about the epidemiological features of HPV in China is critical for global HPV prevention strategies, because of the need to understand the geographic diversity and age distribution of HPV infections. Although a pilot project aimed to measure the hrHPV infection rate and cervical cancer screening was conducted in a few Chinese areas in 1999 [14], and several similar larger scale investigations were carried out that covered more regions that were conducted in 2003, 2008, 2009, and 2012 [15–18], the available data remain insufficient [9] and outdated. Obtaining a more current dataset will also provide a reference for effective screening and vaccination. Herein, we describe a nationwide cross-sectional and large-scale study that had the following aims: 1) to reveal the prevalence of HPV in regions not yet investigated; 2) to follow-up potential changes in HPV infection in regions that have been previously studied; 3) to clarify the genotypic distribution of HPV in different regions and age-grouped populations. These data should expand the data available regarding HPV-related cervical cancer and aid in future research, screening, and vaccination efforts.

## Methods

### Ethics Statement

This study was approved by the Ethics Committee of Tianjin Medical University in accordance with the Ethical Principles for Biomedical Research Involving Human Subjects (Ministry of Health of the People’s Republic of China) and Declaration of Helsinki for Human Research of 1974 (last modified in 2000). Samples were originally obtained from clinical settings for laboratory diagnoses. After diagnostic testing, excess samples were anonymized and kept for this study. Informed consent was obtained from each female participant. For those individuals younger than 18 years old, the consent form was signed by the parents of each participant.

### Study population

**Fig. 1:**
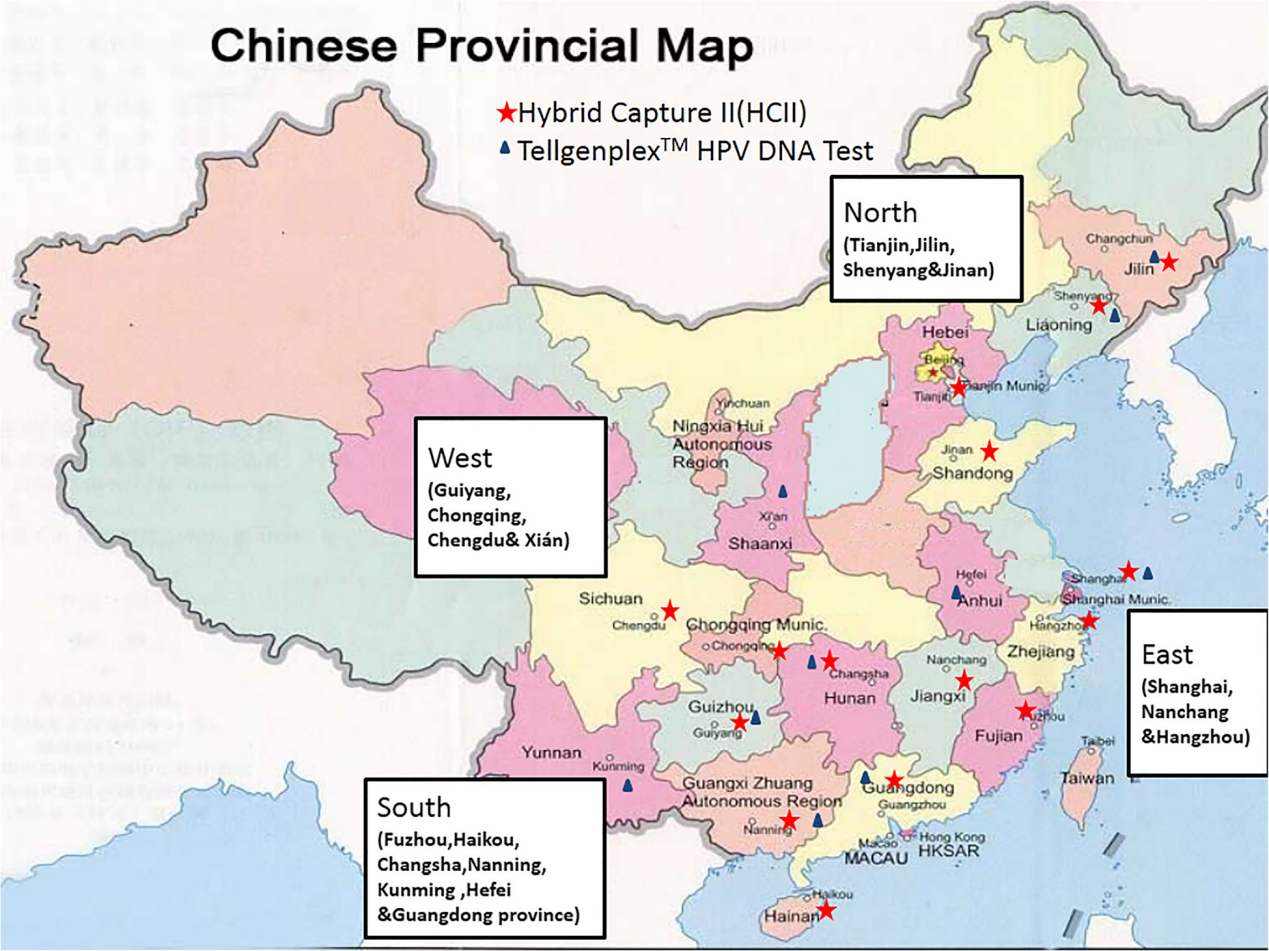
National map of China showing all the geographical sites included in this study

KingMed Diagnostics is the largest reference laboratory in China and provides diagnostic testing services for over 13,000 hospitals in 18 provinces and 4 municipalities across the nation. From January to December of 2012, a total of 120,772 samples were obtained for population- or employee-based screening from 37 cities, belonging to 18 regions (Guangdong province was regarded as one region as 20 cities in this investigation were located in that province). Fig. 1 shows a map of all of the geographical sites in China and the 18 regions were further grouped into 4 macro-geographical regions (East, West, South, and North).

The women enrolled from population- or employee-based cervical cancer screening programs ranged in age from 15 to 60 years old, were sexually active, and had no history of cervical treatment before the screening. Exclusion criteria included current pregnancy, <3 months post-partum, HIV-seropositivity, and a history of either hysterectomy or treatment for cervical cancer.

Among all of the samples, 111,131 were collected from 15 regions *(Haikou, Chongqing, Jinan, Jilin, Shenyang, Tianjin, Shanghai, Nanning, Guangdong, Guiyang, Fuzhou, Hangzhou, Chengdu, Changsha, and Nanchang*; Fig. 1) and were analyzed using Hybrid Capture II (HCII); 105,069 (94.5 %) of these samples could be grouped by age. The other 9641 samples from 10 regions (*Shanghai, Guiyang, Xi’an, Guangdong, Nanning, Changsha, Hefei, Kunming, Shenyang, and Jilin*; Fig. 1) were genotyped using the Tellgenplex™ HPV DNA Test and age data were available for 9,194 (95.4 %) samples.

### Specimen collection

Following currently accepted protocols of practice, cervico-vaginal cells at the transformation zone of the uterine cervix were collected by a gynecologist or a trained gynecologist assistant with a standard cytobrush (with a spatula), then were resuspended in a standard transport medium (STM) and stored at 4 °C [19]. All specimens were coded without knowledge of the subjects. Subsequently, all samples were shipped to the lab of KingMed Diagnostics for HPV testing within 24 h.

### Hybrid Capture II (HCII)

Liquid-based samples were processed by following the instructions included in the Digene sample conversion kit. The hrHPV DNA panel of 13 pooled types (*HPV16, HPV18, HPV31, HPV33, HPV35, HPV39, HPV45, HPV51, HPV52, HPV56, HPV58, HPV59, and HPV68*) was examined using the HCII HPV DNA test (Digene Corporation, Gaithersburg, MD, USA). Data were calculated as a ratio of mean relative light unit (RLU) for the sample to the mean RLU values for the assay of a positive calibrator (PC). A RLU-to-PC ratio >1 (~1.08 pg DNA/ml) was defined as a positive result [20, 21].

### Tellgenplex™ HPV DNA Test

HPV genotyping was performed using the Tellgenplex™ HPV DNA Test (Tellgen Life Science Co., Shanghai, China). The assay can be used to detect and genotype 26 HPV genotypes including 19 hrHPV genotypes (*HPV16, HPV18, HPV26, HPV31, HPV33, HPV35, HPV39, HPV45, HPV51, HPV52, HPV53, HPV55, HPV56, HPV58, HPV59, HPV66, HPV68, HPV82,* and *HPV83*) and 7 lrHPV genotypes (*HPV6, HPV11, HPV40, HPV42, HPV44, HPV61,* and *HPV73*). The Tellgenplex HPV™ kit was used to simultaneously examine the presence or absence of the most common 26 HPV genotypes in a single test by multiplex PCR combined with Luminex technology [22, 23]. The three steps of DNA extraction, PCR amplification, and hybridization were included in the procedure and a template of 10–20 pg/ml HPV DNA was needed for each assay.

### Statistical analyses

#### Region-specific prevalence of HPV

The HPV infection rate in each region was calculated by dividing the number of HPV-positive samples by the total number of samples that were successfully tested for HPV. A binomial 95 % confidence interval (95 % CI) was estimated for each calculation of the prevalence of HPV. Chi-squared (*χ*^2^) tests were used to compare differences among all regions and every two regions.

#### Age-specific prevalence

The HPV infection rate was estimated within 5 age groups (15–19, 20–29, 30–39, 40–49, and 50–60). A binomial 95 % confidence interval (95 % CI) was estimated, and P-values for age trends of HPV infection were analyzed using the linear-by-linear association test. Differences between each pairing of two age groups were compared by the chi-squared (*χ*^2^) test. Multiple comparisons were performed using the Bonferoni step-down procedure to minimize an inflated risk of type 1 error [24]; *P* < 0.05 was considered statistically significant.

#### HPV-type-specific prevalence

The frequency of each hrHPV and lrHPV genotype was presented in hrHPV-positive samples and lrHPV-positive samples, respectively.

All statistical analyses were conducted using SPSS20.0 software (SPSS lnc., Chicago, IL, USA).

## Results

### The total prevalence of hrHPV infection

The total hrHPV infection rate was 21.07 % (95 % Cl 20.83–21.31 %). The prevalence of hrHPV infection differed significant among the various regions (*P* < 0.001). The regions with the highest hrHPV prevalence were Haikou (31.94 %) and Chongqing (27.29 %). By contrast, Nanchang, Changsha, Hangzhou, Chengdu, Fuzhou, Guangdong, and Guiyang could be grouped into the low prevalence regions (Table 1).

**Table 1:** Region-specific prevalence of hrHPV infection by HCII

Regions	Positive samples	Total samples	Infection	95 % CI of infection
			rate (%)	rate (%)
Haikou	221	692	31.94	28.47–35.41
Chongqing	191	700	27.29	23.99–30.59
Jinan	2,651	10,306	25.72	24.88–26.57
Shenyang	98	387	25.32	20.99–29.65
Jilin	360	1,423	25.3	23.04–27.56
Tianjin	807	3,220	25.06	23.57–26.56
Shanghai	118	522	22.61	19.02–26.20
Nanning	1,976	8,869	22.28	21.41–23.15
Guiyang	597	2,919	20.45	18.99–21.91
Guangdong	14,567	72,763	20.02	19.73–20.31
Fuzhou	441	2,213	19.93	18.26–21.59
Chengdu	375	1,886	19.88	18.08–21.68
Hangzhou	649	3,269	19.85	18.49–21.22
Nanchang	290	1,574	18.42	16.51–20.34
Total	23,413	111,131	21.07	20.83–21.31

HrHPV infection rate was different among all the regions using chi-squared test (*P* < 0.001). Subsequently, multiple comparisons were performed using the Bonferoni step-down procedure: marginal differences between the two most heavy-burdened cities of Haikou and Chongqing (*P* = 0.057), and no difference was found among the second group cities, including Jinan, Shenyang, Jilin, and Tianjin (*P* = 0.892), as well as the low infection rate group that included Nanchang, Changsha, Hangzhou, Chengdu, Fuzhou, Guangdong, and Guiyang (*P* = 0.758)

**Fig. 2:**
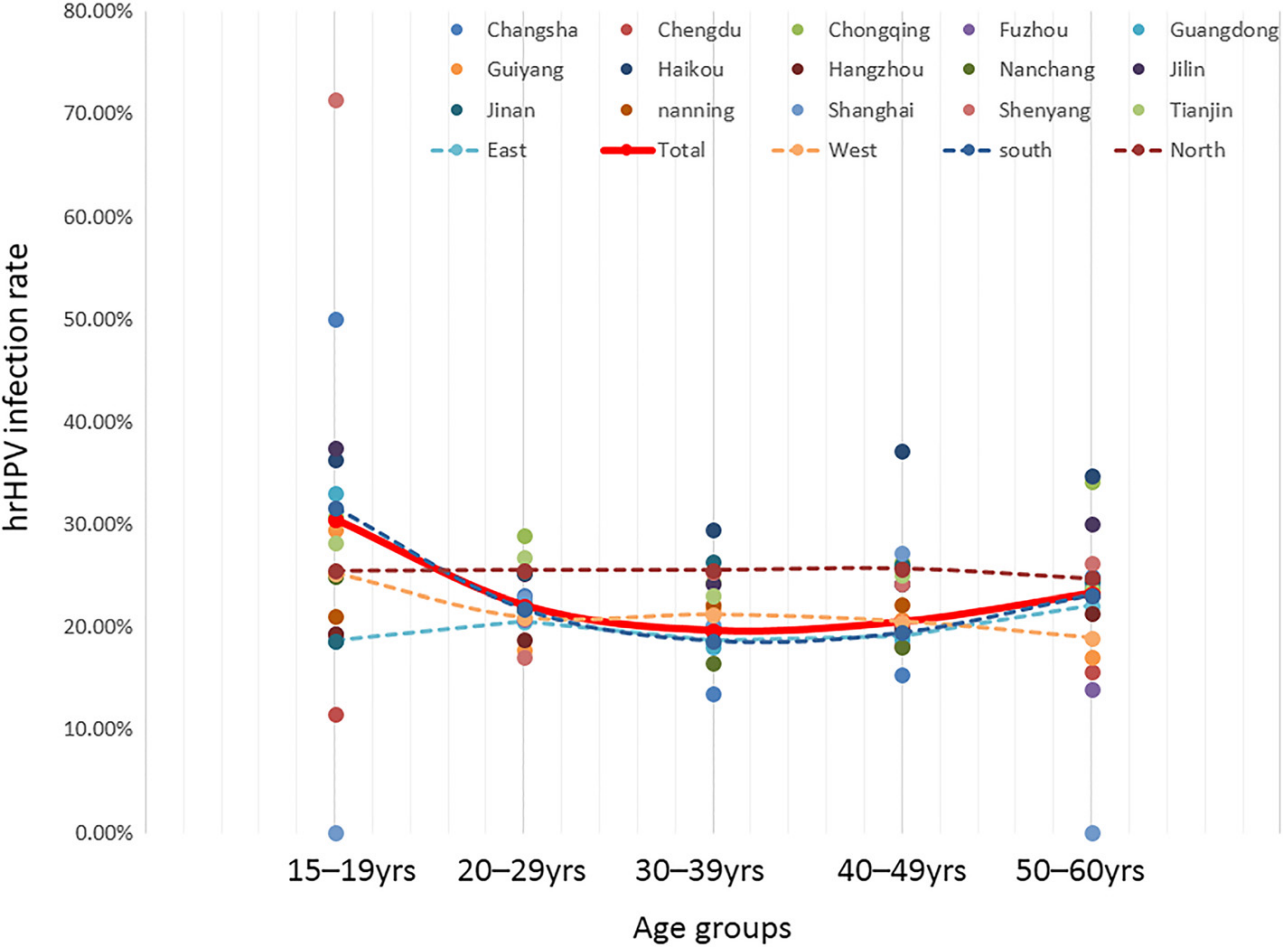
Age-specific hrHPV infection by HCII

As shown in Fig. 2, hrHPV infection was associated with age. The group of 15–19-year-olds showed the highest prevalence (30.55 %), followed by the 50–60-year-olds (23.30 %). The total infection rate of hrHPV was associated with age (*P* < 0.001, Additional file 1: Table S1). After the highest peak, the prevalence declined, but then the infection rate significantly increased again in the 50–60-year-old group compared with the 40-49-year-old group (*P* < 0.001, Additional file 1: Table S1). Among the four macro-regions, only the south showed a similar trend with the overall age-specific hrHPV prevalence, whereas the other three macro-geographic regions did not show significant differences among all five age groups (Fig. 2, Additional file 1: Table S1).

### The distribution of variant HPV genotypes

Among the well-recognized 26 HPV genotypes, all genotypes except HPV73 were examined using the Tellgenplex technique. The total infection rate of each genotype among all of the samples and the distributions of each genotype in the HPV-positive individuals were respectively evaluated. In descending order, the infection rate and distribution are shown in Table 2: the most common hrHPV types were HPV16 (18.02 %) and HPV52 (16.9 %) (*P* = 0.288), moreover, HPV6 (45.80 %), HPV11 (26.15 %), and HPV61 (14.20 %) were common in lrHPV.

**Table 2:** The prevalence of each HPV genotype by the Tellgenplex™ HPV DNA Test

HrHPV Genotypes	Positive Samples	Infection Rate (in 9,641 total samples) %	Proportion (in 2,580 hrHPV-positive samples) %
HPV16	465	4.82	18.02
HPV52	436	4.52	16.90
HPV58	264	2.74	10.23
HPV59	158	1.64	6.12
HPV56	157	1.63	6.09
HPV39	154	1.60	5.97
HPV18	143	1.48	5.54
HPV68	135	1.40	5.23
HPV51	111	1.15	4.30
HPV33	105	1.09	4.07
HPV31	81	0.84	3.14
HPV66	74	0.77	2.87
HPV82	69	0.72	2.67
HPV55	60	0.62	2.33
HPV53	59	0.61	2.29
HPV45	44	0.46	1.71
HPV35	38	0.39	1.47
HPV83	19	0.20	0.74
HPV26	8	0.08	0.31
LrHPV Genotypes	Positive Samples	Infection Rate (in 9,641 total samples)%	Proportion (in 844 lrHPV-positive samples)
HPV6	387	4.01	45.80
HPV11	221	2.29	26.15
HPV61	120	1.24	14.20
HPV40	46	0.48	5.56
HPV44	46	0.48	5.44
HPV42	24	0.25	2.84

Significant differences among the proportion of all genotypes using the chi-squared (*χ* ^2^) test (*P* < 0.0001). Multiple comparisons were further performed using the Bonferoni step-down procedure, and the proportion of the most common genotypes HPV16 and HPV52 (*P* = 0.288), as well as the proportion of HPV59, HPV56, HPV39, HPV18, and HPV68 (*P* = 0.583) were not significantly different

Regarding the region-specific distribution of hrHPV, the top three genotypes were analyzed in each region. HPV16, HPV58, and HPV52 were dominant in six regions—Guiyang, Xi’an, Guangdong, Nanning, Changsha, and Shenyang—although the orders of the three genotypes showed variation in different regions (Additional file 2: Table S2). However, three different top patterns were observed: HPV16, HPV18, and HPV83 in Shanghai; HPV16, HPV33, and HPV82 in Hefei; HPV16, HPV56, and HPV59 in Kunming; as well as HPV16, HPV52, and HPV58 in Jilin (Additional file 2: Table S2).

For lrHPV, HPV11 was the most common genotype in Shanghai and Kunming, while HPV6 was the most frequent genotype in all of the other regions (Additional file 2: Table S2). The distribution of the top three HPV genotypes was also determined on the basis of age. For hrHPV, HPV16, HPV52, and HPV58 were dominant among all of the age groups, except the group of 15–19-year-olds, in which HPV52, HPV16, and HPV59 were the major genotypes (Additional file 1: Table S3). For lrHPV, HPV6 was the leading genotype in all of the age groups, and the second most commonly detected genotype was HPV11 in the younger age groups (i.e., the groups of 15–19, 20–29, and 30–39), while in the older groups (40–49 and 50–60) HPV61 was the most prevalent lrHPV type (Additional file 1: Table S3).

**Fig. 3:**
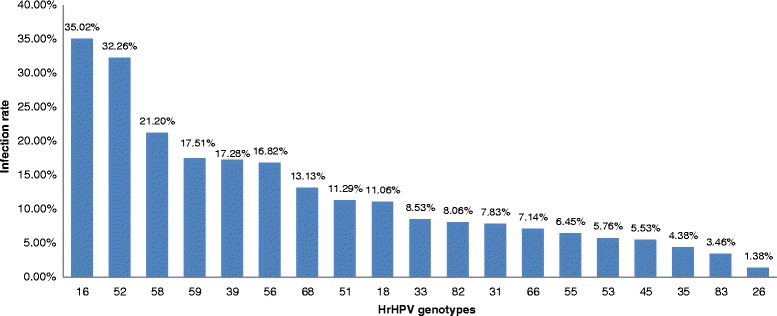
The prevalence of each genotype hrHPV in multiple infections by Tellgenplex™ HPV DNA Test

Infection with multiple HPVs was detected in a total of 486 specimens (5.04 %), among which 434 (16.82 %) and 52 (6.16 %) were infected with hrHPVs and lrHPVs, respectively. In the multiple hrHPV infection individuals, the frequencies of 6, 5, 4, 3, and 2 genotypes were 0.23 %, 1.84 %, 4.61 %, 17.74 %, and 75.58 %, respectively, and three genotypes showed higher positive rates, i.e., HPV16 (35.02 %), HPV52 (32.26 %), and HPV58 (21.20 %) (Fig. 3). The top most common combinations of strains were HPV16 + HPV52 (26 cases) and HPV52 + HPV58 (14 cases) (Additional file 1: Table S3.). For the individuals with multiple lrHPV infections, the proportion of those with 4, 3, and 2 genotypes were 1.92 %, 3.85 %, and 94.23 %, respectively.

**Fig. 4:**
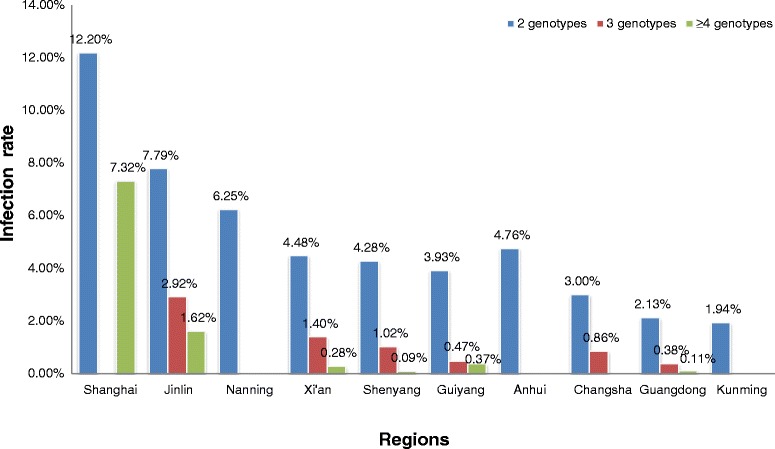
Region-specific multiple infections of hrHPV by Tellgenplex™ HPV DNA Test

Among different regions, the highest incidence of multiple hrHPV and lrHPV infections were in Shanghai (19.51 %) and Nanning (3.13 %), respectively. The distribution of dual, triple, quad and up multiple hrHPV infections in each city showed in Fig. 4. Similar to the overall age trend, of 472 cases with accessible age information, after the first peak in the 15–19-year-olds, another peak was observed in 50–60-year-old individuals both for hrHPV and lrHPV (P < 0.001; Additional file 1: Fig. S1.)

## Discussion

This is one of the few nationwide investigations of high- and low-risk HPV in a large-scale screen of the Chinese population. Because of regional differences, the population composition, and the sampling periods, the reported prevalence varies from study to study, but the most the heavily burdened HPV regions are Sub-Saharan Africa (24.0 %), Eastern Europe (21.4 %), Latin America (16.1 %), and Southeastern Asia (14 %) [25]. In this present survey, the overall hrHPV-positive rate was 21.07 % (95 % Cl 20.83–21.31 %), which increased in levels within HPV-heavy-burden countries and was higher than the average global level.

It is possible that the variable HPV prevalence in China occurs because of the large Chinese population and its territories. Meanwhile, economic conditions, cultural habits, and population migrations have affected Chinese lifestyles and health [11, 15, 26]. Based on population-based screening results that have been previously reported, the overall prevalence of hrHPV varies from 9.9–27.5 % in China [15]. The highest infection rate is in Shanxi, a region with a heavy burden of cervical cancer in China. By contrast, the lowest infection rate was detected in Beijing, the capital of China with prosperous economic and more robust healthcare system. The infection rates in other regions were ~15–20 %, and a pool analysis that included 17 populations from 9 regions showed that the positive rate of hrHPV infection was 17.7 % [27]. Together, the overall hrHPV-positive rate in this present study was found to have increased slightly.

Compared with region-based data, the rates obtained in this present study were higher than those previously reported for Shanghai [28, 29], Shenyang [28], Guangdong [30, 31], and Hangzhou [32] (Additional file 1: Table S5). Additionally, some newly studied regions in this present survey showed high hrHPV incidences, for example HaiKou (31.94 %) and Chongqing (27.29 %), and several cities in the North, including Jinan, Jilin, and Tianjin. These data showed that hrHPV infection is becoming more serious, as the infection rate is increasing in many regions, and some of those regions may soon reach the level of a heavy burden of infection. Notably, great advances in screening strategy and laboratory methods could also have partially contributed to the increased prevalence.

Regarding the hrHPV prevalence in different age groups, a meta-analysis conducted by Bruni et al. [25] showed a bimodal age distribution, with the peak of HPV infection occurring within a younger age group (just after beginning sexual relations). Globally, the lower prevalence of infection in the middle age group was accompanied by a gradual reduction in the incidence in developing countries or a second peak in developed countries [25]. The trend wherein hrHPV infection shows high rates in younger groups and low rates in middle age groups reflects the natural history of HPV infection. Young females are sensitive to HPV soon after beginning of sexual activity because of immature immune protection. Nevertheless, most cases of HPV infection are usually temporary, so in 70 % and 91 % of women infected with HPV, the virus could be cleared within one or two years, respectively [33]. The slight increase in the HPV infection rate in older females might reflect the viral persistence or reactivation of latent HPV, likely because of the physiological and immunological disorders that can result from hormone fluctuations during the menopausal transition [34]. In this study, the general age distribution showed a first peak of hrHPV in the age group of 15-year-old patients (30.55 %), then gradually decreasing in middle age, which is consistent with data from Bruni et al. [25]. However, the hrHPV infection rate was significantly increased in 50–60-year-old individuals compared with women in their 40s, which is a similar trend that has been observed in most developed countries. The prosperous economy in most major cities could influence the culture and sexual behavior of Chinese individuals [15]. Nonetheless, the exact mechanisms for the increase in HPV prevalence still remain unclear.

Data concerning the distribution of HPV genotypes is important for both vaccine development and HPV-based screening design, particularly for selecting the testing spectrum of HPV genotypes [35] and detecting multiple HPV infections. It is a prerequisite for the genotyping assays in cervical cancer screening programs. However, few HPV devices have been cleared by the FDA, including the Roche Cobas 4800, which also provides limited genotyping data [36]. Therefore, the Tellgenplex™ HPV DNA Test, an established genotyping method [23], was performed in this survey. By following CAP (College of American Pathologists) principles, the validation of Tellgenplex™ HPV DNA Test were conducted compare to HCII in the molecular biology lab of KingMed, and the correlations that we detected were found to be (coincident rate = 90.5 %, Kappa = 0.88).

Consistent with the data generated by some Chinese population-specific investigations, HPV16, HPV52, and HPV58 were found to be the dominant hrHPV types [37, 38]. Compared with the data of Wu et al. [28], a population-based investigation from five regions (*Beijing, Shanghai, Xinjiang, Henan,* and *Shangxi*) in China, the infection rates of HPV16 (4.82 %), HPV52 (4.52 %), and HPV58 (2.74 %) were all higher than in the HPV16 (2.9 %), HPV52 (1.7 %), and HPV58 (1.5 %) strains obtained in this present study. Regarding the proportion of these three genotypes, except for HPV52 (16.9 % and 11.9 %, *P* = 0.006), which is higher than that reported in the present study, there were no significant differences detected in the proportion of HPV16 (20.2 % and 18.02 %, *P* = 0.245) or HPV58 (10.8 % and 10.23 %, P = 0.686). The proportion of HPV16 in this survey was even lower than that among the normal cytology samples reported by Guan et al. and Bruni et al. (20.4 % and 22.5 %, respectively) [25, 39]. Notably, HPV52 and HPV58 accounted for 27.13 % of infections, which is markedly higher than the global rate of 14.37 % [39]. Although both HPV52 and HPV58 were all common among Asian populations, the significance of the two genotypes remains unknown. Zhao et al. [40] reported that HPV52 infections are more common among healthy individuals, whereas HPV58 has been linked to cervical cancer. Some studies conducted in the South and West regions of China, which only included CIN or cancer samples, indicated that HPV58 is more prevalent than HPV52 [18, 30, 41, 42]. HPV18, apart from HPV16, is also important for cervical carcinogenesis [43]. However, in our present study, it was the seventh most common infection, and the infection rate (1.48 %) was in line with the study of Wu et al. (*P* = 0.871) [28]. These findings indicated that in addition to HPV16 and HPV18, the HPV vaccine in China should also include the HPV52 and HPV58 genotypes.

In addition to carcinogenesis, many benign cutaneous warts, mucosal lesions, and low-grade cervical intraepithelial lesions generate a considerable health burden that is associated with lrHPV infection [44]. Specifically, HPV6 and HPV11 cause 90 % of genital warts, over 95 % of recurrent respiratory papillomatosis cases, and ~10 % of early cervical lesions [45]. The infection can become more serious in immune-compromised individuals [44]. In our present survey, the incidence of HPV6 (4.01 %) infection was inconsistent with the results of Bruni et al. [25], who showed that HPV6 was most frequent among Americans (2.9 %) and was less frequent in Asian individuals (0.2 %), followed by HPV11 and HPV61.

Characterization of the prevalence of multiple HPV infections might be important for understanding its effects on cervical carcinogenesis. Herrero et al. [10] reported that women infected with HPV16 alone were at a similar or higher risk for cervical cancer than those infected with both HPV16 and another HPV type. Lee et al. [46] reported an association between infection with multiple HPV types and an increased risk of cervical cancer. In a recent study, Schmitt et al. [47] confirmed that co-infection would increase the duration of infection. Furthermore, patients with multiple high viral loads showed a 4- to 6-fold increased risk of cervical precancerous cytological lesions compared with patients with single high viral loads. In this present study, 434 hrHPV-positive samples (4.5 %) were multiple-infections. The incidence was in the same range (3.5–5.3 %) as was previously reported in China [37], but was higher than the global average (3.2 %) [25]. Furthermore, the rate of infection (14.19 %) was lower than reported for both domestic (25.8 %) [28] and international (20 %) [25] populations. The most common combinations of two types were HPV16 + HPV52 (26 cases) and HPV52 + HPV58 (14 cases), and the genotypes HPV52 and HPV58 were more likely to be involved in co-infections. Regarding region-specific surveillance, some geographical features were observed. For example, in Shanghai, a city with a large internal population, the situation was close to the world average regarding HPV prevalence, genotypic distribution, and multiple infections.

This present study confirmed the high overall incidence of HPV in China and strongly argues for the necessity of developing national population-based screening programs. However, the appropriate management of this HPV screening program for a large number of women with HPV-positive specimens and the absence of cytological evidence of cervical pre-cancer or cancer remains a major concern [48]. HPV genotyping could be an option to stratify the HPV-positive women. Furthermore, a stainable HPV detection and close follow-up program for hrHPV carrier women should be implemented. Thus, more cost-effective techniques, such as the Cervista™ HR HPV test, COBAS HPV test, and some other genotyping platforms, might be good alternatives for molecular detection of HPV [49].

## Conclusions

In conclusion, China has large population and a variety of territories; meanwhile, economic conditions, cultural habits, and population migrations have dramatically affected Chinese lifestyle and health, for instance, cancers related to sexual transmitted diseases. Therefore, attention should be paid to the prevalence of HPV infection in a timely and regional basis because it has been commonly recognized that HPV infection plays a critical role in the occurrence of cervical cancer and its increase incidence. These surveillance findings indicated that a national plan for a cervical screening program is urgently needed, not only because of the increase in hrHPV infection rate in some previously reported regions, but also because of the high infection rate in most newly investigated regions. In brief, the most significant findings of this study are as follows: (1) the prevalence of hrHPV infection has reached a level that cannot be ignored, and the rate of increase is growing with time; (2) the prevalence of hrHPV infection showed population variations in age and region, and also reflected economic, cultural, and lifestyle relevance; (3) the HPV16, HPV52, and HPV58 strains consistently constituted the three dominant genotypes in different Chinese populations, a characteristic pattern that was significantly different from the epidemiological features in most of industrial countries. These findings defined principles for future proposals for cervical screening and vaccination in China.

## Additional files

Additional file 1: Table S1. Age-specific HPV infection in each region by HCII. **Table S3. **Age-specific prevalence of each subtype HPV by Tellgenplex™  HPV DNA Test. **Table S4.** The common combinations of double infections by Tellgenplex™ HPV DNA Test. **Table S5. ** HPV Prevalence in Women from population-based Screening Studies. **Figure S1. **Age-specific multiple infection by Tellgenplex™  HPV DNA Test. Additional file 2: Table S2. Region-specific hrHPV and lr HPV subtype prevalence by Tellgenplex™ HPV DNA Test.

## Abbreviations

HPV: Human papillomavirus
hrHPV: High-risk HPV
lrHPV: Low- risk HPV
HCII: Hybrid capture II
CAP: College of American Pathologists
