# Supplementary material for: Nationwide prevalence of human papillomavirus infection and viral genotype distribution in 37 cities in China
Source: BMC Infect Dis. 2015 Jul 4;15:257. doi: 10.1186/s12879-015-0998-5 (PMC4491249; doi:10.1186/s12879-015-0998-5)
Supplement: Additional file 1: Table S1. — Age-specific HPV infection in each region by HCII. Table S3. Age-specific prevalence of each subtype HPV by Tellgenplex™ HPV DNA Test. Table S4. The common combinations of double infections by Tellgenplex™ HPV DNA Test. Table S5. HPV Prevalence in Women from population-based Screening Studies. Figure S1. Age-specific multiple infection by Tellgenplex™ HPV DNA Test. [file 12879_2015_998_MOESM1_ESM.pdf]

Table S1. Age-specific HPV infection in each region by HCII

regions	15-yrs	20-yrs	30-yrs	40-yrs	50-60yrs
**Shanghai**	0.00%	22.81%	20.33%	27.27%	0.00%
**Jiangxi**	25.00%	21.79%	16.60%	18.15%	23.66%
**Hangzhou**	19.44%	18.83%	19.88%	19.82%	21.45%
**East**	**18.75%**	**20.62%**	**18.71%**	**19.25%**	**22.18%**
**Chengdu**	11.63%	22.55%	19.69%	18.32%	15.71%
**Guiyang**	29.52%	17.88%	21.86%	20.86%	17.09%
**Chongqing**	30.77%	29.02%	24.43%	26.35%	34.29%
**West**	**25.29%**	**20.98%**	**21.27%**	**20.59%**	**19.01%**
**Guangdong**	33.11%	21.76%	18.15%	18.85%	23.26%
**Nanning**	21.15%	22.25%	22.22%	22.32%	23.48%
**Hainan**	36.36%	22.13%	29.49%	37.30%	34.85%
**Changsha**	50.00%	23.15%	13.60%	15.46%	25.00%
**Fuzhou**	19.44%	20.91%	20.08%	20.13%	13.95%
**South**	**31.67%**	**21.81%**	**18.72%**	**19.51%**	**23.19%**
**Tianjin**	28.30%	26.78%	23.19%	25.10%	23.67%
**Jilin**	37.50%	25.30%	24.21%	24.28%	30.16%
**Jinan**	18.64%	25.34%	26.36%	26.18%	24.40%
**Shenyang**	71.43%	17.11%	25.42%	24.19%	26.32%
**North**	**25.51%**	**25.58%**	**25.58%**	**25.73%**	**24.81%**
**Total**	**30.55%**	**22.17%**	**19.71%**	**20.55%**	**23.30%**

In Total. P value for age trend of HPV infection was analyzed by using the linear-by-linear association test (P<0.001). Furthermore, Infection rate is difference in each age group in addition to the margin difference in the age group of 20-yrs and 50-60yrs (P=0.051).

In the Four marco-geographic regions, in addition to the South(p<0.001), the other three including the East, West and North were all no significant.(P=0.401, 0.595, 0.974) in each age group.

Table S3. Age-specific prevalence of each subtype HPV by Tellgenplex™ HPV DNA Test

Hr HPV ≤20 (463 cases)				21-30(3253 cases)				31-40(2843 cases)				41-50(2090 cases)				≥51(545 cases)			
Type	Positive	Infection rate	frequency	Type	Positive	Infection rate	frequency	Type	Positive	Infection rate	frequency	Type	Positive	Infection rate	frequency	Type	Positive	Infection rate	frequency
26	0	0.00%	0.00%	83	3	0.09%	0.30%	26	2	0.07%	0.32%	26	0	0.00%	0.00%	26	1	0.18%	0.66%
35	2	0.43%	0.82%	26	4	0.12%	0.40%	45	7	0.25%	1.11%	35	4	0.19%	0.87%	35	2	0.37%	1.32%
83	2	0.43%	0.82%	45	17	0.52%	1.72%	83	7	0.25%	1.11%	45	5	0.24%	1.08%	83	2	0.37%	1.32%
31	4	0.86%	1.65%	55	17	0.52%	1.72%	35	10	0.35%	1.59%	82	5	0.24%	1.08%	45	3	0.55%	1.97%
33	5	1.08%	2.06%	35	18	0.55%	1.82%	66	10	0.35%	1.59%	83	5	0.24%	1.08%	53	3	0.55%	1.97%
53	5	1.08%	2.06%	53	19	0.58%	1.92%	55	14	0.49%	2.22%	51	7	0.33%	1.52%	66	3	0.55%	1.97%
55	5	1.08%	2.06%	82	28	0.86%	2.83%	31	16	0.56%	2.54%	66	12	0.57%	2.60%	82	3	0.55%	1.97%
39	6	1.30%	2.47%	66	33	1.01%	3.34%	53	17	0.60%	2.70%	53	13	0.62%	2.82%	31	4	0.73%	2.63%
45	9	1.94%	3.70%	68	35	1.08%	3.54%	82	19	0.67%	3.02%	55	14	0.67%	3.04%	59	4	0.73%	2.63%
82	10	2.16%	4.12%	31	36	1.11%	3.64%	51	22	0.77%	3.49%	31	18	0.86%	3.90%	33	5	0.92%	3.29%
66	13	2.81%	5.35%	33	37	1.14%	3.74%	59	23	0.81%	3.65%	56	21	1.00%	4.56%	51	5	0.92%	3.29%
18	16	3.46%	6.58%	18	55	1.69%	5.56%	33	29	1.02%	4.60%	59	21	1.00%	4.56%	55	6	1.10%	3.95%
51	17	3.67%	7.00%	51	56	1.72%	5.66%	56	30	1.06%	4.76%	33	23	1.10%	4.99%	56	6	1.10%	3.95%
56	17	3.67%	7.00%	39	66	2.03%	6.67%	18	31	1.09%	4.92%	68	26	1.24%	5.64%	18	8	1.47%	5.26%
68	17	3.67%	7.00%	59	74	2.27%	7.48%	39	32	1.13%	5.08%	18	27	1.29%	5.86%	39	9	1.65%	5.92%
58	24	5.18%	9.88%	56	77	2.37%	7.79%	68	41	1.44%	6.51%	39	33	1.58%	7.16%	68	9	1.65%	5.92%
59	26	5.62%	10.70%	58	82	2.52%	8.29%	58	78	2.74%	12.38%	58	55	2.63%	11.93%	58	15	2.75%	9.87%
16	32	6.91%	13.17%	52	158	4.86%	15.98%	52	114	4.01%	18.10%	52	84	4.02%	18.22%	52	31	5.69%	20.39%
52	33	7.13%	13.58%	16	174	5.35%	17.59%	16	128	4.50%	20.32%	16	88	4.21%	19.09%	16	33	6.06%	21.71%
Total	243	52.48%	100.00%	total	989	30.40%	100.00%	Total	630	22.16%	100.00%	total	461	22.06%	100.00%	Total	152	27.89%	100.00%

LrHPV

6	67	14.47%	51.15%	6	196	6.03%	48.51%	6	66	2.32%	44.90%	6	28	1.34%	37.33%	6	15	2.75%	31.91%
11	39	8.42%	29.77%	11	118	3.63%	29.21%	11	33	1.16%	22.45%	11	13	0.62%	17.33%	11	9	1.65%	19.15%
40	7	1.51%	5.34%	40	23	0.71%	5.69%	40	10	0.35%	6.80%	40	4	0.19%	5.33%	40	1	0.18%	2.13%
42	3	0.65%	2.29%	42	8	0.25%	1.98%	42	6	0.21%	4.08%	42	2	0.10%	2.67%	42	2	0.37%	4.26%
44	1	0.22%	0.76%	44	13	0.40%	3.22%	44	10	0.35%	6.80%	44	11	0.53%	14.67%	44	7	1.28%	14.89%
61	14	3.02%	10.69%	61	46	1.41%	11.39%	61	22	0.77%	14.97%	61	17	0.81%	22.67%	61	13	2.39%	27.66%
Total	131	28.29%	100.00%	Total	404	12.42%	100.00%	Total	147	5.17%	100.00%	Total	75	3.59%	100.00%	Total	47	8.62%	100.00%

Table S4. The common combinations of double infections by Tellgenplex™ HPV DNA Test

Type1	Type2	Positive cases
HPV16	HPV52	26
HPV 52	HPV 58	14
HPV16	HPV59	13
HPV 52	HPV 39	11
HPV16	HPV58	10
HPV 16	HPV 33	10
HPV 52	HPV 68	9
HPV16	HPV56	8
HPV16	HPV39	7
HPV16	HPV68	7
HPV16	HPV51	7
HPV 16	HPV 66	6
HPV 52	HPV 59	6
HPV 52	HPV 18	6
HPV 58	HPV 56	6
HPV16	HPV18	5
HPV 16	HPV 82	5
HPV 52	HPV 56	5
HPV 58	HPV 18	5
HPV 58	HPV 33	5
HPV 58	HPV 66	5
HPV 59	HPV 56	5
HPV 39	HPV 68	5
HPV 39	HPV 53	5
HPV 68	HPV 31	5

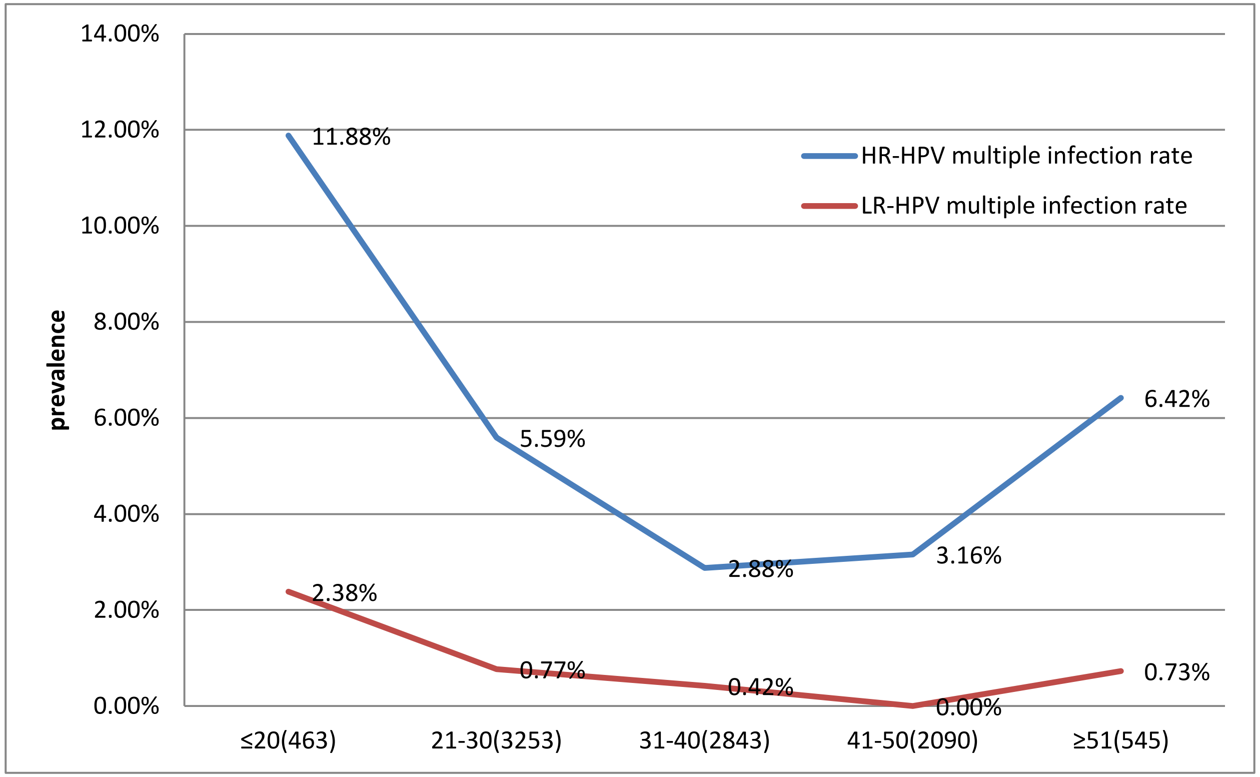

FigS1. Age-specific multiple infection by Tellgenplex™ HPV DNA Test

Table S5 . HPV Prevalence in Women from population-based Screening Studies

Author /Pub year	Study year	Location	Number(N)	Age Range (yrs)	Lab-assays	overall HPV prevalence (%)	
(Belinson et al.2001)	1999	Xiangyuan	1,997	35-45	HC2	18.2	
(Zhao et al., 2001)	2001-2002	Xiangyuan and yangcheng,shanxi	8,798	35-50	HC2	23.6	
(Shen et al., 2003)	2001-2002	Xiangyuan and yangcheng,shanxi	9,683	30-50	HC2	27.5	
(Zou et al., 2011)	2004	Yangcheng	745	15-59	HC2	16.0	
(Li et al., 2007)	2004	Xiushui, Jiangxi	2,432	30-49	HC2	18.5	
(Li et al., 2006)	2004-2005	Shenyang	685	15-59	GP5+/6+ mediated PCR	16.8	
(Dai et al., 2006)	2004-2005	Yangcheng	662	15-59	GP5+/6+ mediated PCR	14.8	
(Wu et al., 2007)	2004-2005	Shenzhen	1,027	15-59	GP5+/6+ mediated PCR	16.6	
(Zhao et al., 2012b)	1999-2008	Shanxi, Beijing(18.8), Xinjiang(8.2), Henan(13.03), Shanghai(18.66)	13,004	16-54	HC2	17.7	
(Li et al., 2010)	2006-2009	Beijing	6,185	25-54	HC2	9.9	
(Ye et al.,2010	2007-2008	zhejiang	5,058	20-79	PCR (MY09/11 primer)	13.3	
(Hu et al., 2011)	2009	Jiangsu	316	18-25	HC2	17.1	
		Shanghai					
(Zhang et al.,2013)	2011	Shanghai suburb	10,000	17-89	PCR (MY09/11 primer)	12.6	

Compared with the region-based data, the results obtained in the present study are higher than those previously reported for Shanghai (this cohort & previously: 22.61% & 18.66% in urban and 12.6% in suburbs)(21, 22), Shenyang (25.32% & 16.8%)(21), Guangdong (20.02% & 16.6% in urban and 9.03% in rural)(23, 24) and Hangzhou (19.85% & 13.3%)(25).
